# Three are better than one: plasminogen receptors as cancer theranostic targets

**DOI:** 10.1186/2162-3619-2-12

**Published:** 2013-04-17

**Authors:** Patrizia Ceruti, Moitza Principe, Michela Capello, Paola Cappello, Francesco Novelli

**Affiliations:** 1 Center for Experimental Research and Medical Studies (CeRMS), Azienda Ospedaliera Città della Salute e della Scienza, Via Cherasco 15, Turin, 10126, Italy; 2 Department of Molecular Biotechnology and Health Science, University of Turin, Turin, Italy

**Keywords:** Annexin 2, Cytokeratin 8, Alpha-enolase, Plasminogen system, Plasminogen receptors, Cancer, Immunotherapy

## Abstract

Activation of plasminogen on the cell surface initiates a cascade of protease activity with important implications for several physiological and pathological events. In particular, components of the plasminogen system participate in tumor growth, invasion and metastasis. Plasminogen receptors are in fact expressed on the cell surface of most tumors, and their expression frequently correlates with cancer diagnosis, survival and prognosis. Notably, they can trigger multiple specific immune responses in cancer patients, highlighting their role as tumor-associated antigens. In this review, three of the most characterized plasminogen receptors involved in tumorigenesis, namely Annexin 2 (ANX2), Cytokeratin 8 (CK8) and alpha-Enolase (ENOA), are analyzed to ascertain an overall view of their role in the most common cancers. This analysis emphasizes the possibility of delineating new personalized therapeutic strategies to counteract tumor growth and metastasis by targeting plasminogen receptors, as well as their potential application as cancer predictors.

## Introduction

The plasminogen system is involved in tumor growth, invasion and metastasis [1-5]. Several proteins of this system have now been demonstrated to have a clinical value as diagnostic and prognostic markers in many types of cancer [6-11]. Overexpression of plasminogen receptors has been associated with poor prognosis, shorter patient survival and resistance to chemotherapy. Notably, this over-expression may lead to the production of autoantibodies in many types of cancers, which can be consequently used as tumor biomarkers. Moreover, most plasminogen system components are cell surface-expressed and therefore represent readily accessible targets for cancer therapy. This review aims to analyze the plasminogen receptors Annexin 2 (ANX2), Cytokeratin 8 (CK8) and alpha-Enolase (ENOA) in the most common tumors and to correlate their expression with specific immune responses and/or clinical outcomes.

### The plasminogen system

Plasminogen is a 90kDa glycoprotein produced by the liver and present in plasma and extracellular fluids [12-15]. It possesses seven structural domains: an N-terminal activation peptide (1–77 aa), five kringle domains and a serine-protease domain (562–791 aa). The kringle domains mediate plasminogen binding to substrates and to cell surface receptors [13,16] whereas the activation of plasminogen to plasmin is mediated by the proteolytic action of either tissue-type (tPA) or urokinase-type (uPA) plasminogen activators [2,4,17,18]. Plasmin is a serine protease with a broad-spectrum substrate, including fibrin, extracellular matrix components (laminin, fibronectin) and proteins involved in extracellular matrix degradation (matrix metalloproteinases, such as MMP3) [16,19-22]. Binding of plasminogen to surface receptors has profibrinolytic consequences: enhancement of plasminogen activation, protection of plasmin from its inhibitor a2-antiplasmin and enhancement of the proteolytic activity of cell-bound plasmin [4,23-25]. Proteolysis mediated by cell-associated plasmin contributes both to physiological processes, such as tissue remodeling and embryogenesis, and to pathophysiological processes, such as cell invasion, metastasis and inflammatory responses [2-4,26-28]. A noteworthy positive correlation exists between elevated levels of plasminogen activation and malignancy [2,29].

Plasminogen receptors bind plasminogen by carboxy-terminal lysines, and this common recognition motif results in a similar affinity of plasminogen for most of its receptors (Kd ≈ 1μM). Although many plasminogen receptors have been described so far [30-43], the most well-known plasminogen receptors shown to play a role in cancer are ANX2, CK8 and ENOA. Interaction of the plasminogen lysine binding sites with ENOA and CK8 is dependent upon recognition of three C-terminal lysines [23,40]. For ENOA, an additional internal plasminogen binding site, which includes Lys256, has been proposed [44]. On the other hand, ANX2 harbors internal amino acids that mimic C-terminal lysines and therefore requires cleavage prior to binding plasminogen [45].

### Molecular features of plasminogen receptors

ANX2 is a 36 kDa peripheral membrane protein (p36) that belongs to the Annexin family, consisting of calcium-dependent phospholipid-binding proteins with various membrane-related functions [46-48]. ANX2 either exists as a monomer, a heterodimer or a heterotetramer, the latter being composed of two copies of a 36 kDa heavy chain (p36) and two copies of an 11 kDa light chain (p11). Formation of the Anx2 heterotetramer facilitates its binding to the plasma membrane [45,48,49].

CK8 is an intermediate filament protein that polymerizes with CK18 to form a component of the epithelial cytoskeleton [50]. In addition to its cytoplasmic localization it can also be expressed on the cell surface [51,52] where it is localized to the blebs of the cell membrane and acts as a plasminogen receptor [53].

ENOA (2-phospho-D-glycerate hydrolase) was initially discovered as a metalloenzyme that catalyzes the dehydration of 2-phospho-D-glycerate (PGA) to phosphoenolpyruvate (PEP) in the glycolytic pathway [54,55]. In vertebrates, the enzyme has three different isoforms (alpha, beta, and gamma) codified by three independent loci. While ENOA is mostly ubiquitous, beta- and gamma-enolase are almost exclusively found in muscle and in neuronal tissues, respectively [54,56,57]. ENOA can form homo- or heterodimers, such as alpha-alpha, alpha-beta and alpha-gamma [58,59]. Both homo- and heterodimers can be expressed on the cell surface where they act as plasminogen receptors.

Interestingly ANX2, CK8 and ENOA amino acid sequences lack a transmembrane portion, and the mechanism by which they are displayed on the cell surface remains unknown. Several mechanisms have been proposed for the cell surface expression, including lipid binding followed by translocation to the outer membrane [60]; penetration and projection through the plasma membrane as a part of a protein complex [52,61]; and non-covalent association to the cell membrane after proteolytic release from cells into the extracellular space [62-64]. In the case of ANX2 and ENOA, post-translational modifications, such as acylation, acetylation, methylation and phosphorylation, particularly common in tumor cells, may also play a role [65-67].

### Expression and function of ANX2 in cancer

ANX2 is physiologically expressed on the surface of epithelial cells, vascular endothelial cells and macrophages and is not only involved in plasminogen activation but also in many biological processes that include inflammation, cell proliferation, angiogenesis, cell–cell interactions and membrane bridgings, exocytosis and endocytosis, cell growth and apoptosis [48,68-73]. All these functions are due to its ability to interact with actin filaments in the cytoskeletal system, to locate proteases and other extracellular matrix components (plasminogen-plasmin-tPA) on the cell surface through tetramerization, or by acting as a major substrate for phosphorylation and as a second messenger of growth-mediating receptor [49]. Indeed, ANX2 is phosphorylated on Tyr23, Ser11 and Ser25 residues by c-Src, v-Src and Protein Kinase C (PKC), respectively [74-76] after activation of insulin receptor [77], insulin growth factor regulator [78], platelet-derived growth factor-R [79], fibroblast growth factor (FGF) or epidermal growth factor (EGF) [80]. Therefore, ANX2 may play a role as a second messenger for transduction of growth and differentiation.

During oncogenic transformation, ANX2 is usually up-regulated and is a marker of aggressiveness in the majority of cancer types (Table 1) [68,81-101]. As a result, it is a valuable tool for cancer diagnosis. In fact, in hepato-cellular carcinoma (HCC), ANX2 has been proposed as a differential diagnostic marker, in combination with glypican-3 (GPC3), glutamine synthetase (GS) and heat shock protein 70 (HSP70) [65,85,86,90,93,98,100-104]. ANX2 down-regulation has also been reported in certain cancers, such as laryngeal and esophageal squamous cell carcinoma, head and neck dysplasia [105-109], osteosarcoma [110] and prostate cancer [111-113] (Table 1). In osteosarcoma and oral carcinoma, ANX2 overexpression is generally associated with well-differentiated tumors and, in certain cases, ANX2 down-regulation was observed in poorly-differentiated cancer which may be due to its ability to promote differentiation independently from its plasminogen-binding function [107,110]. Indeed, in oral carcinoma two different groups have reported an increased expression of ANX2 in tumor tissue compared to the surrounding normal mucosa or mucosa from patients without oral cancer [100,107]. Rodrigo et al. also added a clinical correlation analysis in which the lower expression of ANX2 seemed to correlate with a poor prognosis. Even if these studies appear controversial, both groups reported that in poorly differentiated tumors, which usually correlate with a bad prognosis, ANX2 was down-regulated. We can therefore speculate that ANX2 is up-regulated even in oral carcinoma, but microenvironment and inflammatory responses related to the anaplastic condition induce down-regulation of ANX2 during cancer progression. Opposite trends reported ANX2 as a suitable prognostic marker in HCC, renal (CRCC), colorectal and gastric carcinomas, where its expression negatively correlates with a favorable clinical outcome [85,93,96,100,101]. In fact, when up-regulated, ANX2 contributes to cancer invasion and metastasis by acting as a co-receptor for plasminogen, tPA and pro-cathepsin B [49]. ANX2-dependent plasmin generation is required for invasion, metastasis and angiogenesis in several cancer types [47,65,68,114-117]. For example, it has been demonstrated that in pancreatic cancer (PDAC), tyrosine 23 phosphorylation is necessary for cell-surface localization of ANX2. This translocation is critical for the Transforming Growth Factor beta (TGFbeta)-induced, Rho-mediated epithelial-to-mesenchymal transition (EMT) [65]. Moreover, in PDAC, ANX2 overexpression is a predictor of rapid recurrence after surgery in patients who have undergone gemcitabine-adjuvant chemotherapy [104]. In conclusion, we can hypothesize that in tumors where ANX2 is overexpressed, its role as a plasminogen receptor predominates, and is responsible for the metastatic process, while in tumors where ANX2 is down-regulated, plasminogen-independent mechanisms favor the malignant state through anaplastic transformation.

**Table 1:** Expression, immune response, clinical correlation and function of ANX2 in cancer

**Cancer tissue**	**Expression**	**Immune response**	**Clinical correlation**	**Function**
Bone	protein [110]			Metastasis [110]
Brain	protein [97,98]		Diagnosis [98]	
Breast	mRNA [99]			Chemoresistance, Growth, Metastasis [99,114-116,118]
Colon	mRNA, protein [86,89,96]		Prognosis [86]	
Gastric	protein [85]		Prognosis [85]	
Head and neck	mRNA, protein [100][105-109]	Ab [119,120]	Prognosis [100]	
Hematopoietic system	mRNA [95] protein [68,91]			Growth, Metastasis [68,91]
Kidney	protein [84,101]		Survival, Prognosis [101]	Metastasis [121]
Liver	mRNA, protein [88,90,92,93]		Diagnosis, Prognosis [90,93,103]	
Lung	protein [82]	Ab [122]		Growth, Metastasis [117]
Ovary		Ab [123]		
Pancreas	mRNA, protein [81,87]	Ab [65,124]	Survival, Prognosis [65,104]	Chemoresistance, EMT, Metastasis [65,104]
Prostate	protein [102,111-113]		Prognosis [102]	Growth, Metastasis [47,102]
Skin		T cells [125]		
Thyroid	mRNA [83,94]			

### Expression and function of CK8 in cancer

Several mechanisms have been proposed for the expression of CK8 on the cell surface. In transformed cells, surface expression could be due to insufficient incorporation into intermediate filaments due to over-production of CK8 [64]. Indeed, CK8 is over-expressed both at the mRNA and the protein level in various carcinomas (Table 2) and is present at the cell surface of several human cancers and established tumor cell lines [51,52,64,88,126-129]. By contrast, healthy human tissues do not express CK8 at the cell surface, with the exception of a sporadic and weak CK8 membrane localization in the liver and heart [127,130].

**Table 2:** Expression, immune response, clinical correlation and function of CK8 in cancer

**Cancer tissue**	**Expression**	**Immune response**	**Clinical correlation**	**Function**
Breast	protein [52,64,127,131]	Ab [132]	Prognosis, Survival, Diagnosis [131,133]	Chemoresistance, Growth, Metastasis [134-136]
Colon		Ab [137]		
Gastric	mRNA [128]	Ab [138]		
Head and neck	protein [51]	Ab [51,139]		
Liver	mRNA, protein [52,88]	Ab [140,141]	Prognosis [130]	
Lung	mRNA, protein [126,129]		Survival [126,142]	
Skin				Metastasis [143]
Uterus			Prognosis [144]	Metastasis [144]

When present on the cancer cell surface, CK8 binds plasminogen and promotes its activation through plasminogen activators. In breast cancer cells it also works as a receptor for uPA [134], suggesting a model in which CK8, in complex with uPA, plasminogen and fibronectin constitutes a signaling platform capable of modulating cell adhesion and growth. Indeed, increased expression of CK8/CK18 has been shown at the invasive front of certain tumors [145]. Of particular note, over-expression of CK8 on the tumor cell surface and in the cytoplasm correlates, both *in vitro* and *in vivo,* with increased invasiveness and metastatic properties [144]. In breast and endometrial cancer, a clear correlation exists between CK8 expression and tumor stage [133,144], and CK8 confers drug resistance to chemotherapeutic treatment in breast cancer cell lines [135,136]. The effect of CK8 over-expression on survival remains controversial as in non-small cell lung carcinomas (NSCLC), increasing serum levels of CK8 were significantly associated with tumor progression and lower patient survival [126,142], whereas breast cancer patients with low expression of CK8 and high expression of Human Epidermal Growth Factor Receptor 2 (HER2) have a higher risk of recurrence and death within 5 years (Table 2) [131].

### Expression and function of ENOA in cancer

ENOA over-expression is associated with tumor development through a process known as aerobic glycolysis or the Warburg effect [146], which determines an increase in anaerobic glycolysis in hypoxic conditions, a very common feature in most solid tumors, but also in the presence of normal levels of oxygen [147-149]. The ENOA promoter contains a hypoxia responsive element [150,151] that leads to self up-regulation at both the mRNA and protein levels (Table 3) [130,152-173]. However, in a small fraction of NSCLC [174], ENOA expression is down-regulated and, interestingly, is a gene that can be homozygously absent in tumors [175]. In PDAC, ENOA is not only up-regulated but is also subjected to specific post-translational modifications, namely acetylation, methylation and phosphorylation which do not occur in normal tissue [67,176].

**Table 3:** Expression, immune response, clinical correlation and function of ENOA in cancer

**Cancer Tissue**	**Expression**	**Immune response**	**Clinical correlation**	**Function**
Brain	mRNA [152]			
Bone	protein [170]		Prognosis [170]	
Breast	mRNA, protein [164,167,177]	Ab [178,179]	Prognosis, Survival [167,178]	Chemoresistance, Metastasis [167,177,178]
Colon	mRNA, protein [152,158,168]			
Gastric	mRNA, protein [152,162,169]	T cells [180]		
Head and neck	mRNA, protein [155,166,171]	Ab [119,120]	Prognosis [166]	Metastasis [166]
Hematopoietic system	protein [160]	Ab [181]		
Liver	mRNA, protein [130,152,156,165]		Prognosis [156,165]	Growth, Metastasis [156]
Lung	mRNA, protein [152,159,172,174,182]	Ab [159,172,178,183,184]	Prognosis, Survival [172,174,178]	
Ovary	mRNA, protein [152,153]			
Pancreas	mRNA, protein [152,154,161,163]	Ab and T cells [154,185,186]	Survival [185]	
Prostate	mRNA, protein [152,187]			
Skin	mRNA [173]	Ab [173,188]		

Moreover, ENOA is expressed on the surface of hematopoietic cells, such as monocytes, T cells, B cells and neutrophils according to their activation status or pathophysiological conditions [54]. ENOA is localized on the cell surface of PDAC, breast and lung cancers [67,154,172,189] (Table 3), while in melanoma and NSCLC it is also secreted by exosomes [190,191]. On cell surfaces, ENOA is part of a multi-protein complex, together with uPA receptor (uPAR), integrins and certain cytoskeletal proteins, collectively known as a *metastasome*, responsible for adhesion, migration and proliferation, as clearly demonstrated in ovarian cancer cells [192]. Moreover, since ENOA interacts with the cytoskeleton in muscle cells, its over-expression is likely to promote migration of tumor cells by providing ATP [29]. Noteworthy, in human follicular thyroid carcinoma cells, retinoic acid causes a decrease in ENOA levels that coincides with their reduced motility [193], while cell surface ENOA is enhanced in breast cancer cells rendered super-invasive following paclitaxel treatment [194].

The prognostic value of ENOA expression has been observed in several tumors (Table 3) [156,165-167,170,172,174,178,185]. In breast cancer, enhanced ENOA expression correlates with a greater tumor size, poor nodal status and a shorter disease-free interval. Higher expression of ENOA increases the risk of longer distance relapse when compared to loco-regional relapse in postsurgical 4-Hydroxytamoxifen-treated Estrogen Receptor-positive breast cancer patients. Therefore, down-regulation of ENOA could be a novel pharmacological approach for overcoming 4-Hydroxytamoxifen resistance in breast cancer therapy [167]. In head and neck cancer and NSCLC, ENOA levels increase with cancer progression and negatively correlate with patients’ overall- and progression-free survival [166]. In HCC, ENOA expression is up-regulated in poorly differentiated tumors compared to well-differentiated ones, and its levels positively correlate with tumor size and venous invasion [156,165]. Even if ENO1 are not currently used in clinical routine, the altered expression or the induced immune response, as show later, may have a prognostic/diagnostic value, especially in combination with gold-standard markers. For example, in PDAC, the detection of serum antibodies against phosphorylated ENOA, in combination with the commonly used serum marker CA19.9, enhances the ability to discriminate between control and cancer patients [185]. Taken together, these findings determine that ENOA is a good biomarker to monitor tumor progression and to predict clinical outcome.

### Plasminogen receptors elicit immune responses in cancer

Activation of the immune system is an early event during tumorigenesis, as illustrated by the detection of high titers of autoantibodies in patients with early-stage cancer, and correlates with the progression of malignant transformation [119,195,196]. The presence of an immune response to the plasminogen receptors ANX2, CK8 and ENOA has been reported in patients with different types of cancer.

An autoantibody response against ANX2 occurs in the early stages of gingivo-buccal carcinogenesis and in PDAC, ovarian, and lung cancer (Table 1) [65,119,120,122,123]. It has been demonstrated that in melanoma, the ANX2 (208–223) peptide induces antigen-specific T cells, which can recognize cancer cells over-expressing the ANX2 molecule. This peptide may therefore be useful in immunotherapy for recruiting CD4+ type 1 helper cells locally active in the tumor environment [125]. Moreover, phase II clinical trial PDAC patients [124] showed an increased anti-ANX2 antibody response after administration of the GM-CSF (granulocyte-macrophage colony-stimulating factor) PDAC-specific vaccine, which positively correlated with prolonged survival [65].

CK8/18 complexes elicit humoral responses in breast, gastric, colon, head and neck and liver carcinomas [51,132,137-141]. In squamous cell carcinoma (SCC), elevated titers of CK8-specific serum antibodies are detectable in early stage patients, while a weaker humoral response has been observed in advanced patients, probably due to immunosuppression by tumor cells. In several adenocarcinomas, patients develop an immune response against tumoral neo-epitopes of CK8 [137], while head and neck and lung cancer patients develop autoantibodies against post-translationally modified variants of the protein (Table 2) [139,142]. The induction of specific antibodies against CK8 can be explained by its ectopic expression on the cancer cell surface that makes CK8 a potential candidate for antibody-based cancer therapy.

A humoral and/or T cell immune response against ENOA has been observed in patients with breast, head and neck, gastric, lung cancer, chronic myeloid leukemia, melanoma and PDAC (Table 3) [119,120,154,159,172,173,178-181,183-186],[188]. Notably, detection of autoantibodies against ENOA combined with expression of carcino-embryonic antigen (CEA) and CK19 fragment enhances the sensitivity for differential diagnosis of NSCLC [184]. High levels of antibodies to phosphorylated ENOA were found in PDAC, and their combination with the serological marker CA19.9 discriminates patients from controls [185]. The presence of the humoral response to phosphorylated ENOA also correlates with a longer progression-free survival upon gemcitabine treatment and overall survival [185] and notably, with the presence of a specific T cell response [154,180,186,197]. Conversely, a marked decrease in basal levels of ENOA-autoantibodies is a common event in late lung and breast cancers, proposing ENOA-autoantibodies as a prognostic marker to monitor disease progression in these patients [178].

### Plasminogen receptors as therapeutic targets in cancer

Despite heterogeneous numbers of plasminogen receptors existing in eukaryotic cells, only ENOA, CK8 and ANX2 have a recognized role in human cancer progression by promoting plasmin-dependent tumor invasion. ANX2 also behaves as a tPA receptor, whilst ENOA and CK8 do not directly bind plasminogen activators, but most likely form a multi-protein complex with uPAR and integrins [64,67]. Nevertheless, the contribution of these three receptors to cancer development is not only linked to their plasminogen-binding capacity. Indeed ANX2, ENOA and CK8 all interact with the actin cytoskeleton and such an association could promote the migration of tumor cells independently from plasminogen-binding [29]. These three receptors are present on the surface of the majority of human neoplasms, and in some cases, such as breast and colon, they are expressed altogether. All of these receptors elicit an antibody immune response in cancer patients that can be potentially boosted for therapeutic purposes. Regarding the potential risk of inducing an autoimmune reaction, CK8 would probably be the best candidate as it is aberrantly expressed only on the tumor cell surface. However, even the ANX2 and ENOA are frequently over-expressed by tumor cells and exposed on the cell surface. This strengthens the hypothesis for utilizing them as potential targets of therapeutic monoclonal antibodies. Notably, data obtained in animal models show a therapeutic effect of anti-ANX2 monoclonal antibody (mAb) treatment in breast, pancreatic and lung cancer by inhibiting tumor growth, suppressing metastases and prolonging survival (Table 1) [65,114,117]. Thus, blocking plasminogen receptors is a promising strategy to counteract metastasis development.

A recent paper has shown that a DNA-based vaccination against human ENOA, followed by electroporation, was able to significantly prolong the median of life expectancy of mice that spontaneously develop PDAC [186]. Indeed, the ENOA DNA vaccine induced both a humoral and cellular response. Autoantibodies against ENOA were able to bind the cell surface of PDAC murine cells and mediate their killing by complement-dependent cytotoxicity. This “immunogenic” death might be responsible for the greater influx of CD3 into tumor and for their activation toward Th1/Th17 phenotype. Cytokines produced by these Th populations strongly supported the “effecto” switching of anti-ENOA autoantibodies [186]. Moreover, the ENOA-DNA vaccine significantly decreased suppressor cells such as myeloid-derived suppressor cells and regulatory T cells [186].

## Conclusions

Overall, with very few exceptions, all three plasminogen receptors reported here display performant diagnostic and prognostic values. Since one single receptor type is not expected to account for the entire plasminogen binding on tumor cells, a combined therapy targeting all these molecules would be desirable. Nevertheless, further studies are necessary in order to better understand the involvement of these three receptors in cancer progression, so that more effective strategies for cancer treatment may be developed.

## Abbreviations

aa: amino acid; ANX2: Annexin 2; CA19.9: Carbohydrate antigen 19.9; CEA: Carcino-embryonic antigen; CK8: Cytokeratin 8; CK18: Cytokeratin 18; CK19: Cytokeratin 19; CRCC: Chromophobe renal cell carcinoma; EMT: Epithelial mesenchymal transition; EGF: Epidermal growth factor; ENOA: Alpha-enolase; FGF: Fibroblast growth factor; GM-CSF: Granulocyte-macrophage colony-stimulating factor; GPC3: Glypican 3; GS: Glutamine synthetase; HCC: Hepato-cellular carcinoma; HER2: Human epidermal growth factor receptor 2; HSP70: Heat shock protein 70; kDa: Kilo-Dalton; Lys: Lysine; mAb: monoclonal antibody; MMP3: Matrix metalloproteinases 3; NSCLC: Non-small cell lung cancer; PDAC: Pancreatic ductal adenocarcinoma; PEP: Phosphoenolpyruvate; PGA: 2-phospho-D-glycerate; PKC: Protein kinase C; SCC: Squamous cell carcinoma; Ser: Serine; TGFbeta: Transforming Growth Factor beta; Th: T helper cell; tPA: tissue-type activator; Tyr: Tyrosine; uPA: urokinase-type activator; uPAR: urokinase-type activator receptor.

## Competing interests

The authors declare that they have no competing interests.

## Authors’ contributions

PC and MP equally contributed to writing the first draft. MC and PC refined the draft version of the manuscript. FN supervised the other contributors and critically revised the manuscript. All authors read and approved the final manuscript.
